# Comparison of the health-related quality of life of end stage kidney disease patients on hemodialysis and non-hemodialysis management in Uganda

**DOI:** 10.1186/s12904-021-00743-0

**Published:** 2021-04-01

**Authors:** Peace Bagasha, Elizabeth Namukwaya, Mhoira Leng, Robert Kalyesubula, Edrisa Mutebi, Ronald Naitala, Elly Katabira, Mila Petrova

**Affiliations:** 1 School of Medicine, Department of Internal medicine, Makerere University College of Health Sciences, P.O. Box 7072, Kampala, Uganda; 2 Makerere-Mulago Palliative Care Unit, Clinical Research Building, Mulago hospital site, P.O. Box 7072, Kampala, Uganda; 3 Baylor College of Medicine Children’s Foundation Uganda, P. O Box 72052, New Mulago Road, Kampala, Uganda; 4 Cambridge Palliative and End of Life Care Research Group, Primary Care Unit, Department of Public Health and Primary Care, University of Cambridge, Cambridge, UK; 5 Cambridge Institute of Public Health, Forvie Site, Cambridge, CB2 0SR UK

**Keywords:** Quality of life [MeSH], Kidney failure, Chronic [MeSH], Palliative care [MeSH], Renal Dialysis [MeSH], Developing countries [MeSH], Resource limited setting, Low and middle income countries, LMIC

## Abstract

**Background:**

Health-related quality of life is recognized as a key outcome in chronic disease management, including kidney disease. With no national healthcare coverage for hemodialysis, Ugandan patients struggle to pay for their care, driving families and communities into poverty. Studies in developed countries show that patients on hemodialysis may prioritize quality of life over survival time, but there is a dearth of information on this in developing countries. We therefore measured the quality of life (QOL) and associated factors in end stage renal disease (ESRD) patients in a major tertiary care hospital in Uganda.

**Methods:**

Baseline QOL measurement in a longitudinal cohort study was undertaken using the Kidney Disease Quality of Life Short Form Ver 1.3. Patients were recruited from the adult nephrology unit if aged > 18 years with an estimated glomerular filtration rate ≤ 15mls/min/1,73m^2^. Clinical, demographic and micro-financial information was collected to determine factors associated with QOL scores.

**Results:**

Three hundred sixty-four patients **(**364) were recruited, of whom 124 were on hemodialysis (HD) and 240 on non-hemodialysis (non-HD) management. Overall, 94.3% of participants scored less than 50 (maximum 100). Mean QOL scores were low across all three principal domains: physical health (HD: 33.14, non-HD: 34.23), mental health (HD: 38.01, non-HD: 38.02), and kidney disease (HD: 35.16, non-HD: 34.00). No statistically significant difference was found between the overall quality of life scores of the two management groups. Breadwinner status (*p* < 0.001), source of income (p0.026) and hemodialysis management type (p0.032) were the only factors significantly associated with QOL scores, and this was observed in the physical health and kidney disease principal domains only. No factors were significantly associated with scores for the mental health principal domain and/or overall QOL score.

**Conclusion:**

The quality of life of Ugandan patients with ESRD has been found to be lower across all three domains of the Kidney Disease Quality of Life Short Form than reported anywhere in the world, with no difference observed between the non-HD and HD management groups. Interventions targeting all domains of QOL are needed among patients with ESRD in Uganda and, potentially, in other resource limited settings.

## Background

The World Health Organization (WHO) defines quality of life (QOL) as “an individual’s perception of their position in life in the context of the culture and value systems in which they live and in relation to their goals, expectations, standards and concerns” [1]. In developed world settings, various studies have demonstrated that QOL is an independent predictor of both hospitalizations and mortality [2–4]. Therefore slowing deterioration or increasing quality of life has progressively become a key outcome of successful disease management, including end stage kidney disease (ESRD).

The global burden of kidney disease is increasing, with incidence in the past three decades rising by 88% (11 to 21 million), prevalence by 87% (147 to 275 million), death by 98% (0.6 to 1.2 million) and disability adjusted life years by 62% [5]. The leading drivers for this have been the global increase in non-communicable diseases, particularly Diabetes Mellitus and Hypertension [5]. Resource-limited settings in Africa, Asia and Latin America [5] face the greatest burden of the global increase in chronic kidney disease (CKD) due to the double burden of communicable and non-communicable disease. In these settings, diarrheal diseases, malaria and HIV are additional important contributors to chronic kidney disease burden [6, 7]. In 2017, CKD caused more deaths globally than did HIV or tuberculosis [8].

In contrast to epidemiological patterns observed in resource-rich settings, chronic kidney disease incidence in resource-limited settings peaks in adolescents and young adults, further limiting economic productivity in an environment which is already little equipped to handle the disease [5]. Without medical management, chronic kidney disease typically progresses to ESRD [9–11]. In resource-limited settings, poor health seeking behaviors and lack of specialist care for renal disease mean that the majority of patients present for the first time having already developed ESRD [12, 13]. In Uganda, the prevalence of chronic kidney disease has been found to vary from 0.7 to 21.4% in community studies [14, 15] while 51% of patients attending a tertiary renal clinic had ESRD, with only 9% in stage 1 CKD [16].

Worldwide, the quality of life of patients with ESRD is typically poor [17, 18]. In resource-rich settings, treatment for patients with ESRD includes hemodialysis (HD), peritoneal dialysis and kidney transplantation [19]. These treatments, in addition to supportive care, such as access to nutritional support, psychosocial supports, spiritual support and optimal medical management of co-morbidities, have been associated with an improved quality of life [20]. In resource-limited settings such as Uganda, where hemodialysis costs at least $400 USD/month and renal transplantation is only available abroad for a cost of at least $30,000 USD [21, 22], the majority of patients with ESRD have no access to adequate healthcare due to lack of health insurance or the personal resources to pay for it.

With less than 20 Nephrologists serving a population of over 45 million Ugandans and dialysis units in two out of 134 districts in the country, the majority of patients have limited access to specialized care. Patients who ultimately access HD often receive one or two sessions per week, with very few managing a third session due to the financial implications. Those who opt for non-HD management receive a limited form of palliative care from generalist care providers, lacking spiritual and psychosocial components but with an emphasis on alleviation of symptoms and referral to care providers closer to the patient’s home. Symptom alleviation for these patients includes control of complications of renal failure such as; volume overload (treated by diuresis and volume restriction); uremic syndrome (treated by intestinal dialysis with laxative induced diarrhea); anemia (blood products and erythropoietin stimulating agents); bone mineral disease (calcium replacement and phosphate binders) and metabolic acidosis (oral bicarbonate replacement). Comprehensive palliative care, however, involves far more than symptom alleviation. It is an approach that provides holistic (physical, psychosocial and spiritual) management aimed at improving the health-related quality of life of patients and families facing life-threatening illness, such as ESRD [18, 23]. Although palliative care services have been available in Uganda since 1993, most of them are provided to cancer and HIV/AIDS patients [24]. Yet ESRD patients in other settings have been found both to have palliative care needs [25] and to benefit from associated interventions [18].

No studies have explored the quality of life or palliative care needs of ESRD patients in Uganda. We undertook a study among ESRD patients in Uganda on HD and non-HD management to measure their quality of life and explore the factors associated with it. The evidence we generate will seek to inform the development of management strategies that could best optimize the quality of life for patients with ESRD in Uganda and other resource-limited settings.

## Methods

### Study design

This study represents baseline measurement in a longitudinal cohort study of patients with ESRD attending the adult nephrology unit of Mulago National Referral Hospital. Data were collected between January and November 2019. Mulago Hospital is a tertiary care centre located in Kampala, Uganda, which houses the largest renal unit in the country. Patients diagnosed with renal failure from all parts of the country, including neighbouring countries like the Democratic Republic of Congo and South Sudan, are often seen in this unit, which consists of a renal outpatient clinic, a renal inpatient ward and an HD unit. Details of the broader study can be found in the protocol paper [26].

### Patient selection

Non-probability consecutive sampling was used to recruit 364 participants using a proportion of 2:1 of non-HD relative to HD patients. We chose this recruitment design because of small overall patient numbers and anecdotal evidence of high morbidity and mortality amongst HD patients in particular. Recruitment was carried out by two trained research assistants. The sample size was calculated to achieve a study power of 80% with 95% confidence level for the comparison of two means – HD vs non-HD, accounting for a high attrition rate set at 30% [27]. To be eligible for participation in the study, patients had to be aged > 18 years with documented evidence of ESRD defined as chronic kidney disease stage V (estimated glomerular filtration rate of 15mls/min/1.73m^2^ or less calculated using Cockcroft-Gault Formula). Patients with acute kidney injury defined as an elevated serum creatinine for a period of less than three months were not eligible for enrolment.

### Data collection

Once patients had given written informed consent, a four-part study questionnaire, pre-tested in a pilot study, was administered to them by two research assistants. The latter were trained (further details in protocol paper [26]), observed while collecting data and found to be capable of creating good rapport with patients and to maintain neutrality while asking questions and filling in patient responses. The pilot study had shown that patient self-administration of the questionnaire resulted into poor quality data.

Part 1 of the questionnaire collected sociodemographic and financial information; Part 2 consisted of the Kidney Disease Quality of Life-Short Form (KDQOL-SF) Version 1.3 and collected quality of life information; Part 3 used The African Palliative Care Association Palliative care Outcome Scale (APCA POS) to collect information on palliative care needs; and Part 4 used the Renal symptoms Palliative care Outcome Scale (POS-S Renal) to score patient symptomatology. This paper reports on data from Parts 1 and 2.

The KDQOL-SF Version 1.3 was developed by the RAND Corporation [28] with multinational validation for use in both resource-rich and resource-limited countries [29–31]. Three principal domains are assessed in the measurement of QOL and presented as composite summary scores: Kidney Disease Composite Summary (KDCS), Physical Composite Summary (PCS) and Mental Composite Summary (MCS). These are further subdivided into 19 subdomains, each comprised of a number of items namely:

- KDCS: Symptom burden − 12 items; Kidney disease effects on daily life − 8 items; Kidney disease burden − 4 items; Cognitive functioning − 4 items; Employment − 2 items; Sexual functioning − 2 items; Social interaction quality − 3 items; Quality of sleep − 4 items; Social support structures − 2 items, Support from dialysis staff − 2 items; and Satisfaction of patient − 1 item.
- MCS; Fatigue/energy − 4 items; Social function − 2 items; Role emotional (role limitations due to emotional functioning) -5 items; and Mental health − 3 items.
- PCS: Physical function − 10 items; Role physical (role limitations due to physical functioning) -4 items; Pain − 2 items; and General health − 5 items.

The Hays algorithm was used to generate mean scores for the three principal domains and their subdomains [20]. The maximum achievable score for any one domain (principal or subdomain), representing the best quality of life, is 100.

As part of this study, the KDQOL-SF tool was translated into Luganda, the most common Ugandan language used in and around Kampala, and adapted for the Ugandan context [26]. Guidelines provided by RAND Health Care were followed during tool translation [32].

### Statistical analysis

Questionnaire data were entered into EpiData Ver. 3.1 [33] and analysed in STATA version 12.0 [34] after final data cleaning. Descriptive statistics were used for summarizing the data on sample composition and characteristics. QOL scores were calculated for each participant following Hays algorithm [28] and then overall scores computed as means and standard deviations. With 100 representing the best QOL score, patient scores were categorised into high (≥50) and low (< 50) for the scale as a whole and for each of the three principal domains (physical, mental and kidney disease specific). Fifty was chosen as a cut-off having been used in QOL studies worldwide [35]. For the Physical health and Mental health principal domains, a score of 50 is standardized to the general public [36, 37].

Continuous variables were reported as either mean ± standard deviation (SD) or median and interquartile range (IQR) based on their distribution. Categorical variables were analyzed using Chi-square test or the contingency coefficient, as appropriate. For the quantitative variables, after checking normality with the Kolmogorov-Smirnov test, the T-Student test or Mann-Whitney were used to compare the scores between hemodialysis (HD) and Non-hemodialysis, as suitable. Simple and multiple linear regression analyses were performed to identify the predictors of KDCS, PCS, and MCS. *P*-values of < 0.05 were considered statistically significant [38]. The variables with *p* < 0.20 at univariate analysis were selected and included in the multivariate linear regression model using backward variable selection method.

## Results

### Demographic and clinical data

**Fig. 1:**
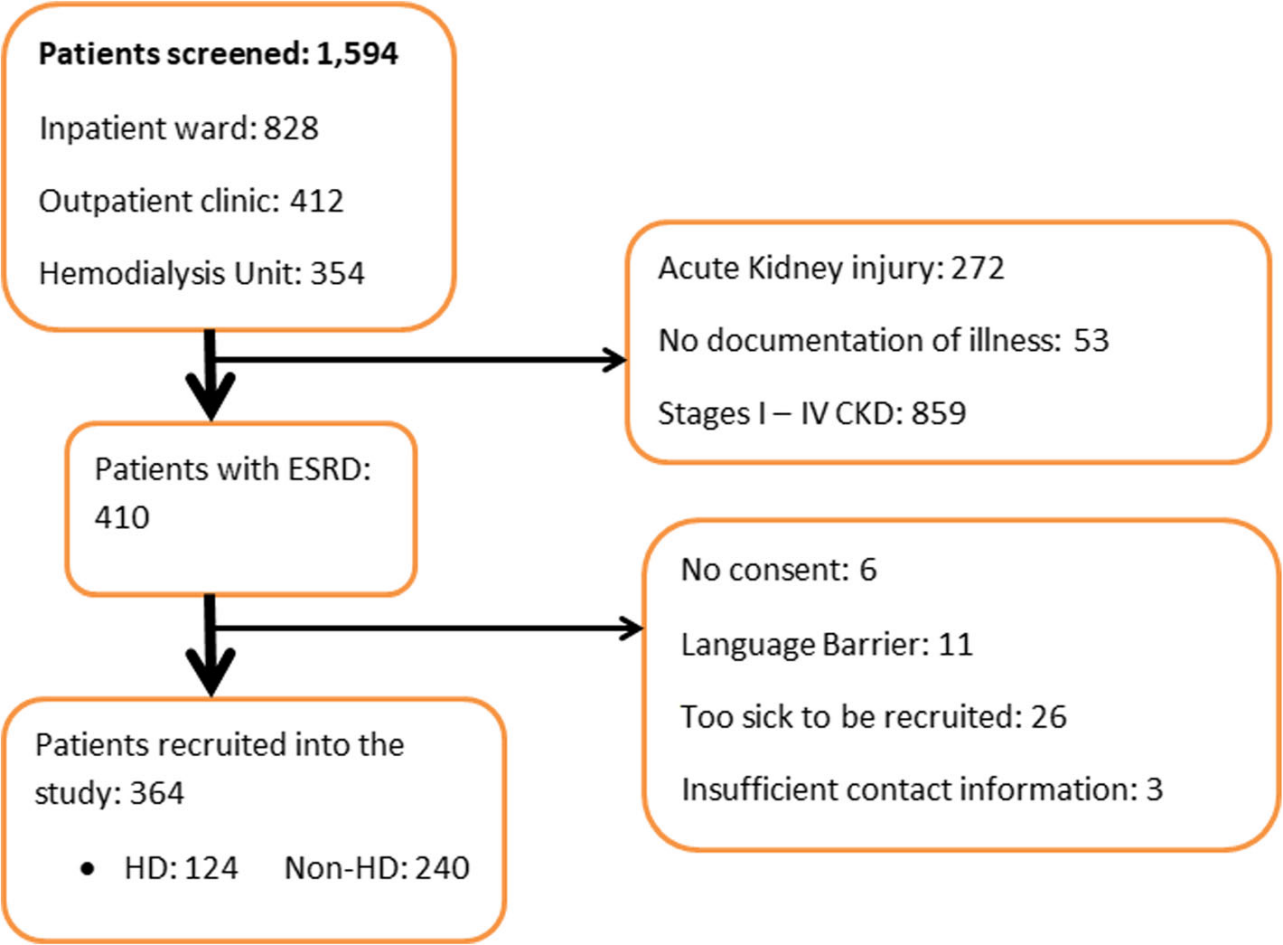
Patient Recruitment Flow chart

One thousand five hundred ninety-four patients were screened from the renal outpatients’ clinic, renal inpatients’ ward and the HD unit from January 2019 to November 2019. Three hundred and sixty four (364) patients were enrolled into the study. The majority of patients were ineligible to participate because they had chronic kidney disease stages I to IV (859/1594) or acute kidney injury (272/1594). Among patients with chronic kidney disease stage V, the commonest reason for ineligibility was severe morbidity (26/410) and language barrier (11/410). Of the recruited patients, 124 were on HD while 240 patients were on non-HD management (Fig. 1). As shown in Table 1, 62% of participants were aged < 50 years; the majority were male (60.2%), married (62.3%), with a family size of ≤5 (56.3%) and belonging to the Christian faith (72.7%). An overwhelming majority reported that they were employed (92%), although only 45.9% reported that their jobs were their main source of income and 27.7% that they were the breadwinners in the family. Hypertension was the commonest comorbidity (80.8%) followed by Diabetes (27.5%). Education level and the kidney disease principal domain scores were the only statistically significant difference between the two patient management groups. The HD group had higher education levels compared to the non-HD group (*p*-value < 0.001) and higher kidney disease principal domain scores (*p*-value < 0.001). Overall QOL scores, however, were not significantly different between the two groups (*p*-value 0.102).

**Table 1:** Characteristics of End Stage Renal Disease Patients in a tertiary hospital in Uganda

Characteristics	Non-Haemodialysis		Haemodialysis		Overall Total (***N*** = 364)		***p***-value
	***n*** = 240	%	***n*** = 124	%			
*Gender*							*0.134*
Female	98	42.6	39	34.2	137	39.8	
Male	132	57.4	75	65.8	207	60.2	
*Age*							*0.294*
18–50	152	63.9	71	58.2	223	62.0	
≥ 50	86	36.1	51	41.8	137	38.0	
Median (IQR)	45 (34,56)		48 (36,58)		46 (35,58)		
*Marital status*							*0.196*
Married	138	57.5	80	64.5	218	62.3	
Single	102	42.5	44	35.5	146	37.7	
*Education level*							<.001*
None	26	10.8	13	10.5	39	10.7	
Primary	78	32.5	19	15.3	97	26.6	
Secondary	99	41.3	46	37.1	145	39.8	
Post-Secondary	37	15.4	46	37.1	83	22.8	
*Family size*							*0.346*
≤ 5	128	58.2	58	52.7	186	56.3	
> 5	92	41.8	52	47.3	144	43.6	
*Religion*							*0.211*
None/Others	15	6.3	13	10.5	28	7.6	
Muslim	42	17.5	29	23.4	71	19.5	
Catholic	97	40.4	43	34.7	140	38.4	
Protestant	86	35.8	39	31.5	125	34.3	
*Occupation*							*0.720*
Employed	220	91.7	115	92.7	335	92.0	
Unemployed	20	8.3	9	7.3	29	8.0	
*Main source of income*							*0.138*
Donations	69	28.7	36	29.0	105	28.8	
My job	103	42.9	64	51.6	167	45.9	
Savings/others	68	28.3	24	19.4	92	25.3	
*Bread winner*							*0.182*
No	168	70.0	95	76.6	263	72.3	
Yes	72	30.0	29	23.4	101	27.7	
*Hypertension*							*0.101*
No	52	21.7	18	14.5	70	19.2	
Yes	188	78.3	106	85.5	294	80.8	
*Stroke*							*0.146*
No	235	97.9	118	95.2	353	97.0	
Yes	5	2.1	6	4.8	11	3.0	
*Diabetes*							*0.609*
No	172	71.7	92	74.2	264	72.5	
Yes	68	28.3	32	25.8	100	27.5	
*KDCs*							<.001*
≤ 50	170	70.8	53	42.7	223	61.3	
> 50	70	29.2	71	57.3	141	38.7	
Median (IQR)	49.1 (38.7, 57.6)		54.7 (48.5, 60.4)		51.1 (41.3, 58.9)		
*Overall health score*							*0.102*
≤ 50	208	92.9	107	97.3	315	94.3	
> 50	16	7.1	3	2.7	19	5.7	
Median (IQR)	40.9 (35.9, 43.9)		42.2 (38.8, 44.5)		41.3 (37.1, 44.2)		

*KDCS* Kidney disease composite summary, *PCS* Physical composite summary, *MCS* Mental composite summary *Statistically significant Variables (*P*-value < 0.05)

### Quality of life score

94.3% of patients had an overall mean QOL score of less than 50 (maximum 100): median of 42.2 (38,8, 44.5) for the HD group and 40.9 (35.9, 43.9) for the non-HD, with the difference between the two groups not statistically significant (Table 1)

Details of the domain and subdomain scores are shown in Table 2. There was no statistically significant difference between the non-HD and HD groups in the three principal domain scores, which were, respectively: PCS of 34.23 vs 33.14, MCS of 38.02 vs 38.01 and KDCS of 48.52 vs 53.04. Overall, Social support at 74.24 (non-HD) and 78.36 (HD) was the highest scoring subdomain for both patient management groups. In contrast, patients identified major role limitations due to both physical health (5.93 non-HD and 0.21 HD) and emotional health (6.07 non- HD and 0.55 HD). These were the lowest scoring items, particularly in the hemodialysis group.

**Table 2:** Quality of Life Domain Scores for Patients with End Stage Renal Disease in a tertiary hospital in Uganda

Parameter	Non-Haemodialysis		Haemodialysis		*p*-value
	Mean	SD	Mean	SD	
**Kidney disease-specific domains**					
Symptom/problem list [12]	34.48	36.36	65.82	24.23	1.0000
Effects of kidney disease [8]	47.85	24.92	47.15	22.54	0.3967
Burden of kidney disease [4]	25.57	17.92	19.25	15.32	0.0005**
Work status [2]	31.88	26.56	45.56	22.99	1.0000
Cognitive function [3]	67.39	22.98	63.66	22.47	0.0699
Quality of social interaction [3]	59.58	16.90	61.88	16.90	0.8902
Sleep [4]	47.17	19.51	42.66	16.78	0.0147**
Social support [2]	74.24	29.12	78.36	29.13	0.8994
**KDCS**	**48.52**	**13.59**	**53.04**	**12.16**	**0.9990**
**SF-36**					
Physical functioning [10]	29.02	26.42	18.89	22.27	0.0002**
Role limitations--physical [4]	5.93	22.75	0.21	2.29	0.0033**
Pain [2]	66.71	27.11	72.94	27.36	0.9791
General health [5]	37.95	15.23	39.66	37.13	0.8453
Emotional well-being [5]	50.82	11.33	52.85	10.37	0.9483
Role limitations--emotional [3]	6.07	22.71	0.55	4.29	0.0044**
Social function [2]	61.56	22.79	59.21	24.95	0.1927
Energy/fatigue [4]	47.93	13.46	50.13	12.51	0.9296
**PCS**	**34.23**	**7.63**	**33.14**	**6.02**	**0.0957**
**MCS**	**38.02**	**5.80**	**38.01**	**4.77**	**0.4866**
**OVERALL HEALTH SCORE**	**40.43**	**6.20**	**41.71**	**4.42**	**0.9733**

**significant at *p* < .05; *KDCS* Kidney disease composite summary, *SF* Short Form health survey, *MCS* Mental composite summary, *PCS* Physical composite summary; Maximum score = 100 per domain, *SD* Standard Deviation Numbers in brackets represent number of items in each subdomain

Statistically significant differences between the scores of the two groups were however noted in five subdomains (Table 2). In the kidney disease principal domain, these were Burden of kidney disease (25.57 non-HD vs 19.25 HD) (*p*-value: 0.0005) and Sleep (47.17 vs 42.66) (p-value: 0.0147). In the Physical health principal domain, these were Physical functioning (29.02 vs 18.38) (p-value: 0.0002) and Role limitations due to physical function (5.93 vs 0.21) (p-value: 0.0033). In the Mental health principal domain, this was Role limitations due to emotional functioning (6.07 vs 0.55) (p-value: 0.0044). In all five subdomains the non-HD group had higher scores reflecting better quality of life.

In univariate analysis (Table 3), we found three factors to be significantly associated with low QOL scores, namely: main source of income (p0.024, CI (− 7.90, − 0.57)), breadwinner status (*p* < 0.001, CI (− 9.76, − 3.80)) and management type (p0.0033, CI (− 3.52, − 0.15)). All three factors were significant in the kidney disease or physical health principal domains, with none significantly associated with overall QOL scores or the mental health principal domain. Hemodialysis management type demonstrated a tendency to higher QOL scores in the kidney disease principal domain only, while savings as the main source of income and not being a breadwinner were associated with lower QOL scores in the kidney disease and physical health principal domains.

**Table 3:** Impact of social and clinical parameters on Quality of life domain scores

Parameter	KDCS			PCS			MCS		
	Beta	***p***-value	95% CI	Beta	***p***-value	95% CI	Beta	***p***-value	95% CI
*Gender*									
Male	− 0.476	0.743	[− 3.33, 2.37]	− 0.869	0.283	[− 2.46, 0.72]	− 0.559	0.377	[− 1.81, 0.69]
*Age*									
30–49	3.142	0.123	[− 0.86, 7.14]	0.849	0.465	[− 1.44, 3.13]	1.588	0.076	[− 0.16, 3.34]
50–70	2.042	0.339	[− 2.15, 6.23]	0.213	0.861	[− 2.19, 2.62]	1.387	0.140	[− 0.46, 3.23]
> 70	3.459	0.341	[− 3.68, 10.60]	−1.115	0.595	[−5.24, 3.01]	− 1.107	0.492	[− 4.27, 2.05]
*Marital status*									
Not Married	−1.821	0.200	[−4.61, 0.97]	−0.705	0.378	[−2.28, 0.87]	− 0.290	0.636	[−1.49, 0.91]
*Education level*									
Primary	2.108	0.402	[−2.83, 7.05]	1.807	0.201	[−0.97, 4.58]	0.448	0.679	[−1.68, 2.58]
Secondary	0.867	0.717	[−3.83, 5.57]	1.646	0.224	[−1.01, 4.30]	0.391	0.706	[−1.65, 2.43]
Post-Secondary	4.404	0.088	[−0.66, 9.46]	2.049	0.156	[−0.78, 4.88]	0.710	0.521	[−1.46, 2.89]
*Family size*									
3–5	1.657	0.419	[−2.37, 5.68]	1.034	0.367	[−1.21, 3.28]	−0.369	0.673	[−2.09, 1.35]
6 +	0.994	0.634	[−3.10, 5.09]	0.684	0.558	[−1.61, 2.98]	−1.338	0.134	[−3.09, 0.41]
*Religion*									
Muslim	−0.274	0.927	[−6.12, 5.58]	−1.863	0.277	[−5.23, 1.50]	−1.374	0.297	[−3.96, 1.22]
Catholic	0.366	0.895	[−5.06, 5.79]	−1.692	0.285	[−4.80, 1.42]	−1.126	0.355	[−3.52, 1.27]
Protestant	−0.577	0.836	[−6.01, 4.90]	0.001	0.999	[−3.15, 3.16]	−1.605	0.194	[−4.03, 0.82]
*Occupation*									
Unemployed	−3.941	0.125	[−8.99, 1.11]	2.237	0.148	[−0.79, 5.27]	0.703	0.553	[−1.63, 3.03]
*Main source of income*									
My job	2.322	0.154	[−0.88, 5.52]	1.470	0.119	[−0.38, 3.32]	0.592	0.416	[−0.84, 2.02]
Savings/others	−4.235	0.024*	[−7.90, −0.57]	−1.042	0.322	[−3.11, 1.02]	−0.380	0.640	[−1.98, 1.22]
*Bread winner*									
No	−6.784	<.001*	[−9.76, −3.80]	−1.841	0.033*	[−3.52, −0.15]	−1.184	0.073	[−2.48, 0.11]
*Hypertension*									
No	0.819	0.643	[− 2.65, 4.29]	−0.731	0.458	[−2.67, 1.20]	0.784	0.298	[−0.69, 2.26]
*Stroke*									
No	0.464	0.909	[−7.54, 8.47]	−1.054	0.681	[−6.09, 3.99]	− 1.464	0.456	[−5.32, 2.39]
*Diabetes*									
No	0.308	0.844	[−2.76, 3.38]	1.229	0.157	[−0.47, 2.93]	−0.957	0.150	[−2.26, 0.348]
*Cancer*									
No	−3.641	0.586	[−16.79, 9.50]	−0.777	0.829	[−7.87, 6.31]	1.613	0.559	[−3.81, 7.04]
*Type of patients*									
Haemodialysis	4.448	0.003*	[1.50, 7.39]	−0.897	0.285	[−2.55, 0.75]	0.069	0.916	[−1.22, 1.36]

*significant at *p* < .05; *KDCS* Kidney disease composite summary, *SF* Short Form health survey, *MCS* Mental composite summary, *PCS* Physical composite summary

In multivariate analysis (Table 4), main source of income, breadwinner status and management type were again identified to contribute significantly to participants’ QOL. Hemodialysis management (p0.032, CI (0.29, 6.32)) and the patient’s job being their main source of income (p0.026, CI (0.27, 4.29)) were associated with higher QOL scores in, respectively, the kidney disease and physical health principal domains, while not being a breadwinner was associated with lower scores (p0.001, CI (− 9.48, − 3.02)) in the kidney disease principal domain.

**Table 4:** Multiple linear regression analysis of factors associated with low Quality of life scores

Parameter	KDCS			PCS			MCS		
	Beta	***p***-value	95% CI	Beta	***p***-value	95% CI	Beta	***p***-value	95% CI
*Gender*									
Male	−0.119	0.938	[−3.12, 2.88]	−0.735	0.400	[− 2.45, 0.98]	− 0.447	0.519	[−1.81, 0.92]
*Education level*									
Primary	0.854	0.778	[−5.11, 6.82]	2.017	0.236	[−1.32, 5.35]	1.741	0.198	[−0.91, 4.39]
Secondary	−0.343	0.907	[−6.13, 5.44]	2.025	0.223	[−1.24, 5.29]	1.668	0.206	[−0.92, 4.26]
Post-Secondary	2.050	0.513	[−4.11, 8.21]	2.564	0.144	[−0.88, 6.01]	1.961	0.159	[−0.78, 4.69]
*Occupation*									
Unemployed	−4.150	0.128	[−9.49, 1.19]	1.213	0.454	[−1.97, 4.39]	0.405	0.753	[−2.12, 2.93]
*Main source of income*									
My job	2.896	0.100	[−0.56, 6.35]	2.279	0.026*	[0.27, 4.29]	0.981	0.228	[−0.62, 2.58]
Savings/others	−1.199	0.542	[−5.06, 2.67]	0.093	0.933	[−2.09, 2.28]	0.128	0.885	[−1.61, 1.87]
*Bread winner*									
No	−6.253	<.001*	[−9.48, −3.02]	−1.678	0.070	[−3.49, 0.13]	−1.023	0.164	[−2.46, 0.42]
*Stroke*									
No	1.741	0.664	[−6.13, 9.62]	−1.429	0.575	[−6.44, 3.58]	−1.075	0.595	[−5.06, 2.90]
*Diabetes*									
No	0.381	0.806	[−2.67, 3.43]	1.363	0.122	[−0.37, 3.09]	−1.072	0.126	[−2.45, 0.30]
*Type of patients*									
Haemodialysis	3.306	0.032*	[0.29, 6.32]	−1.193	0.176	[−2.92, 0.54]	−0.157	0.822	[−1.53, 1.22]

*significant at *p* < .05; *KDCS* Kidney disease composite summary, *SF* Short Form health survey, *MCS* Mental composite summary, *PCS* Physical composite summary

## Discussion

We measured the health-related quality of life of patients with end stage renal disease in Uganda, using the KDQOL-SF Ver 1.3, and compared the scores of patients on hemodialysis to those on non-hemodialysis management. We also explored factors associated with quality of life scores in both management groups. The scores of our sample of Ugandan ESRD patients were lower than the scores of any group of ESRD patients reported in the literature we have accessed, with no statistically significant difference between the two management groups. Breadwinner status, source of income and management type were the only factors significantly associated with QOL scores.

### Patient characteristics in context

The mean age, 45.9 years, of our study population was comparable to that in other studies from developing countries such as India (42, +/− 13.4) [35], Ghana (43, +/− 17.8) [39] and Nigeria (42, +/− 15.43) [40] but different from that in studies from developed countries such as the United Kingdom (82, +/_6) [25], USA (62, +/_14) [3] and the Netherlands (> 70) [41]. This reflects the varying aetiologies of kidney disease in different settings but also demonstrates that, in the developing world, the impact of kidney disease is borne by the most economically productive age groups, with adverse consequences for development. Additionally, the younger patient age – indicating better physiological capability and, therefore, a higher likelihood for better long term outcomes – ends up losing its relative advantages due to seriously limited resources to cover healthcare related expenditure.

Males (at 60.2%) were the predominant sex in our study, a recurrent pattern in the majority of studies from both developed and developing counties such as Ghana (64.5%) [39], USA (59%) [3] and India (68.1%) [35]. In our study, it matched our experience-based observations of the health seeking behaviours of Mulago Hospital patients. As a tertiary care centre, Mulago Hospital is a setting of expensive care. Women may prefer to avoid referral due to healthcare related factors (e.g. distance from their family and support systems and lack of personal financial capacity to cover healthcare related expenditure) or patient related factors (e.g. low educational level, preference for alternative medicine and concerns about voicing their pain and suffering) [42]. Similar findings have been reported in a Nigerian study where 59.04% of patients accepted hemodialysis because they could afford it, with only 20.48% of these patients being female [22]. We also found that 72% of participants were not the breadwinners in the family and 51.64% of these were males, implying that many females are left with the burden of caring for the patient and providing for large families (41%, > 5 individuals) because of loss of income from the spouse. This contributes to the rising trend of household financial distress associated with chronic disease in families [21, 22, 43].

Patients on HD were significantly more likely to have a higher educational level compared to patients on non-HD management. This is a reflection of the inequity in access to renal replacement therapies based on socioeconomic status that has been described in various studies [44–46]. Developed settings have broader hemodialysis access in comparison to developing settings [46] and educated patients are more likely to afford and sustain treatment, especially where no or limited health insurance exists [45].

### Health related quality of life findings in context

Our findings showed that overall mean HRQOL for patients with ESRD is low, with 94.3% participants scoring less than 50 (out of 100) and only 19 participants scoring over 50. The scores obtained in this study were lower than any previously reported KDQOL-SF scores, both in developed and developing world settings. An Indian study reported physical composite scores of 31.76 (in HD patients) and 30.36 (in non-HD patients), mental composite scores of 42.24 (HD) and 42.39 (non-HD), and kidney disease composite scores of 60.45 (HD) and 60.51 (non-HD) [35]. Of these, only the physical composite scores were lower than those in our study, namely of 33.14 (HD) and 34.23 (non-HD). Moreover, the kidney disease composite scores we recorded were 7 to 12 points lower than in the Indian sample, namely of 53.04 (HD) and 48.52 (non-HD). The difference in the mental composite scores was more moderate, with figures from the current study of 38.01 (HD) and 38.02 (non-HD). Furthermore, a systematic review of studies on QOL of hemodialysis and peritoneal dialysis (PD) patients from seven middle or high income countries (South Africa, Saudi Arabia, Ireland, Korea, Brazil, Singapore and USA) estimated higher pooled QOL scores: physical composite scores of 39.50 (PD) and 40.00 (HD), mental composite scores of 47.50 (PD) and 46.50 (HD) and an overall health related quality of life score of 63.00 for both PD and HD [47].

It can be argued that the uniquely low quality of life scores amongst Ugandan ESRD patients result, to a significant degree, from resource limitations, presenting as lack of access to a plethora of support services available in resource-rich countries, such as nutritional advice or psychotherapy; lack of supportive institutional policies, such as free access to Erythropoetin stimulating agents or complimentary transportation to and from the hemodialysis units; and, most importantly, lack of access to kidney transplant programs.

### Comparison of QOL between hemodialysis and non-hemodialysis management groups

We found no statistically significant difference in overall quality of life scores for the two management groups. However, there were statistically significant differences between the scores of the two groups in five subdomains, namely: Burden of kidney disease, Sleep, Physical functioning, Role limitations due to physical functioning and Role limitations due to emotional functioning. In all of these subdomains, patients on non-HD management scored higher. A recent systematic review of four studies comparing the QOL scores of the two group types showed higher mental composite scores and a sustained higher overall HRQOL for patients on non-HD as opposed to higher physical composite scores for patients on HD which, however, declined over time [48]. The study authors suggested that the coping strategies employed by the non-HD group, such as acceptance of the disease and adjustment to life with their disease condition as well as the impending end of life, ultimately lead to better HRQOL. Additionally, reports from developed settings indicate that HD may not provide a clear benefit over non-HD in terms of survival and quality of life, especially for an aging population and in the presence of extensive comorbidities [49, 50].

Our hospital has only one nutritionist for the entire 800-bed facility and no dedicated physiotherapist or psychologist covering the renal units. Patients spend heavily on transport and rented accommodation, especially if they live far from the hemodialysis unit. In order to reduce expenditure, patients on hemodialysis routinely carry out only two hemodialysis sessions per week, while international standards stipulate a minimum of three sessions per week [51]. As a result, the clearance of uremic toxins can be inadequate, with implications for symptom burden and, in turn, quality of life, contributing to the minimal overall difference found between the QOL of patients on hemodialysis and those on non-HD. This too may contribute to the elimination of the physical health benefit of dialysis seen in other studies [48].

The lack of routine assessments of health-related quality of life in our setting may be a further factor explaining the low scores found. In the US, for example, patients receiving hemodialysis by the Centre for Medicare Services should be offered a mandatory routine HRQOL assessment within four months of dialysis initiation and thereafter annually or as necessitated after any significant life changing event [52]. Studies from developed settings have shown improvements in quality of life in patients for whom exercise regimens or programs have been introduced into dialysis sessions, but this and other related interventions have to be triggered by routine assessment of baseline HRQOL [53]. Routine assessment of depression is also potentially important for achieving improvements in health-related quality of life, as depression has been found to contribute to low mental composite scores and, in one study, to be associated with increased risk for death and hospitalization [54, 55].

### Factors associated with QOL scores

We found only three factors to be significantly associated with QOL scores: source of income, breadwinner status and management type. A patient’s job being their main source of income was associated with a tendency towards higher QOL scores in the physical health domain. Hemodialysis management was associated with higher QOL scores and not being a breadwinner was associated with lower QOL scores, both observed only in the kidney disease principal domain. No factors were found to be significantly associated with scores for the mental health principal domain and/or overall QOL score.

Similarly to our findings, which suggest that a patient’s job being their main source of income was associated with higher QOL scores, a Taiwanese multicenter study found that higher monthly income or health insurance were positively associated with health-related quality of life in hemodialysis patients [56]. Studies from Nepal and South Africa have shown hemodialysis management to be associated with higher QOL scores in the physical health principal domain while we found it to be associated with higher QOL scores in the kidney disease domain [57, 58]. This may be due to different degrees of physical symptom alleviation achieved by adequate vs. inadequate number of dialysis sessions.

### Strengths and limitations

Our study has been the first to explore the quality of life of end stage renal disease patients in Uganda. It used an internationally validated and widely used instrument for assessing HRQOL in patients with ESRD, adding credibility to its findings and offering opportunities for comparisons with findings from other settings. The tool was translated into one of the local languages of Uganda, culturally adapted to the local setting and validated (paper in preparation). To our knowledge, this is also the study with the largest participant sample assessing quality of life in patients with ESRD in Sub-Saharan Africa. Sample sizes in other studies ranging from 22 to 202 participants, namely 22 in a study from Malawi [59], 106 in a study from South Africa [60] and 202 from Ghana [12].

Findings may have limited generalizability by virtue of being generated in a single centre. At the same time, this is the largest renal unit in the country and part of the national referral hospital, receiving patients from the whole of Uganda as well as neighbouring countries, such as the Democratic Republic of Congo, Rwanda and South Sudan. The hospital is also serving a significant refugee population from Ethiopia, Somalia, Burundi and the above neighbouring countries.

We found only three variables to be statistically significantly associated with quality of life – not being the breadwinner, hemodialysis treatment and patient’s job as the main source of income – but the relationship was only limited to the kidney disease specific domain (the former two variables) and the physical health principal domain (the third variable). They had no significant association with overall QOL. It is possible that the pattern of scores we observed – too low across the board and thus lacking variety – has precluded the identification of valid risk factors. A different tool or method may be needed to trace risk factors in our particular setting.

As this was a baseline study, it is also possible that participants have not had enough time to adjust to their clinical diagnosis. The ongoing follow-up quantitative study and accompanying qualitative interviews will enable us to draw a more accurate picture of how patients adjust to their illness, what coping strategies they employ and, ultimately, what it is like to live with ESRD in Uganda.

## Conclusion

Patients with ESRD in Uganda are younger than in developed countries, predominantly male, and have significantly compromised quality of life scores compared to those reported from other developed or developing settings. There was no significant difference in overall QOL scores between patients on hemodialysis in comparison to those on non-hemodialysis management. HD was positively associated with kidney disease composite scores but does not appear to improve the overall HRQOL of patients with ESRD in this setting. It is associated with an increased kidney disease burden, poor sleep, reduced physical functioning and role limitations due to changes in physical and emotional functioning.

## Data Availability

The authors have full control over the primary data. As per the research ethics committee approval, this dataset is subject to ethical restrictions and local data protection regulations that do not allow publication of raw data. All relevant data for the conclusions are presented in the manuscript**.**

## Abbreviations

CKD: Chronic Kidney Disease
ESRD: End Stage Renal Disease
HD: Hemodialysis
KDQOL-SF: Kidney Disease Quality of Life short form
QOL: Quality of life
